# Quiet eye facilitates sensorimotor preprograming and online control of precision aiming in golf putting

**DOI:** 10.1007/s10339-016-0783-4

**Published:** 2016-11-07

**Authors:** Joe Causer, Spencer J. Hayes, James M. Hooper, Simon J. Bennett

**Affiliations:** Research Institute of Sport and Exercise Sciences, Liverpool John Moores University, Byrom Street, Liverpool, Merseyside L3 3AF UK

**Keywords:** Preprograming, Online control, Feedback, Aiming, Perceptual-cognitive skill

## Abstract

An occlusion protocol was used to elucidate the respective roles of preprograming and online control during the quiet eye period of golf putting. Twenty-one novice golfers completed golf putts to 6-ft and 11-ft targets under full vision or with vision occluded on initiation of the backswing. Radial error (RE) was higher, and quiet eye was longer, when putting to the 11-ft versus 6-ft target, and in the occluded versus full vision condition. Quiet eye durations, as well as preprograming, online and dwell durations, were longer in low-RE compared to high-RE trials. The preprograming component of quiet eye was significantly longer in the occluded vision condition, whereas the online and dwell components were significantly longer in the full vision condition. These findings demonstrate an increase in preprograming when vision is occluded. However, this was not sufficient to overcome the need for online visual control during the quiet eye period. These findings suggest the quiet eye period is composed of preprograming and online control elements; however, online visual control of action is critical to performance.

## Introduction

Vickers (1992) was one of the first to examine expertise differences in gaze control during a golf putting task. Low-handicap golfers were reported to use a strategy consisting of longer fixations on the ball, with fewer fixations on the putting surface or club. Conversely, the high-handicap golfers fixated all the locations a similar amount. Furthermore, when comparing shot success, a stable fixation on the ball during the swing, and then on the putting surface after contact, was associated with an increased probability of success. In a follow-up study, Vickers (2004) found that poor putters had a variable gaze pattern relative to the ball, whereas good putters kept a stable fixation on the back of the ball during the swing, along with a more stable and deliberate scan path between the ball and the hole. Taken together, these data provide evidence that a long, stable fixation during the golf putt increases the probability of success (Vickers 2007).

Based on the work above and other seminal research on the role of gaze behaviours in aiming tasks (Vickers 1996a), Vickers coined the term ‘quiet eye period’ to describe the final fixation on a specific location before initiation of action (for a review see: Wilson et al. 2015). In golf putting, it is thought that an effective quiet eye period consists of: (1) a single, long, continuous fixation on the back of the ball; (2) an onset before backswing; (3) a continued fixation through the backstroke, forestroke and contact; (4) a dwell time after contact (Vickers 2007; Vine et al. 2013). It is suggested that prioritising task-relevant visuo-spatial information for skill execution during the final fixation leads to a reduction in cortical resources associated with analytical processing and attention to irrelevant sensory cues (Vickers 2009). As such, the quiet eye period appears to functionally represent the time spent processing visual information responsible for motor control (Vickers 1996c).

Since its conception, the quiet eye period has been predominantly associated with the preprograming of action (Vickers 2011), which in golf putting would equate to movement organisation occurring before the initiation of the putter swing without visual or proprioceptive feedback influencing the action (Vickers 2007). In support of the preprograming hypothesis, it has been shown that experts exhibit a prolonged quiet eye period and greater cortical activation in the right-central region (i.e. Bereitschaftspotential) compared to non-experts (Mann et al. 2011). The suggestion is that prolonged fixations, particularly during the final fixation that defines the quiet eye period, permit the detailed processing of information and cortical organisation necessary for preprograming effective motor performance. As a result, longer quiet eye durations have been associated with more efficient and less variable movement kinematics that require fewer online modifications (Causer et al. 2010, 2011). Further support for the preprograming hypothesis is provided by Williams et al. (2002), who examined the relationship between quiet eye and task complexity in billiards. Based on the widely accepted principle that more complex or difficult motor responses (e.g. aiming to a far vs. near target, Fitts 1954) require longer preprograming time (Henry 1980; Klapp 1977), Williams et al. (2002) reported longer quiet eye durations for more complex shots, thus implying the role of programming during the quiet eye period.

While not intending to downplay the role of preprograming during the quiet eye period, there is some suggestion that the process of online visual control might also be involved (Wilson et al. 2015). In golf putting, online control equates to the use of visual and/or proprioceptive feedback to adjust the movement after initiation (Vickers 2007). This idea is not new, but has received very little research attention. Vickers (1992) found that on successful shots, and for skilled golfers, the final fixation extended through the foreswing and beyond contact. Subsequently, it has been found that a quiet eye dwell time of around 250 ms, which is the period from putter–ball contact until gaze offset, is most effective for a successful golf putt (Vickers 2007). More evidence of online control was reported by Vine et al. (2013), who found that unsuccessful golf putts had a reduced quiet eye duration between initiation of backswing and ball contact (QE-online), as well as from putter–ball contact to gaze offset (QE-dwell); no changes in quiet eye duration during the period before backswing (QE-preprograming) were found. The implication is that the change of gaze location was detrimental to online visual control processes involved after movement initiation. Such results are consistent with the importance of online control and utilisation of late visual information to regulate actions requiring a high level of precision (Craig et al. 2000; Oudejans et al. 2002). Based on these studies, it would seem advantageous in golf putting to maintain fixation on the ball as this facilitates processing of visual information from the putter as it is moved away (backswing) and then towards (foreswing) the ball.

A study by Vine et al. (2015) examined the contribution of preprograming and online control during the quiet eye period in golf putting by occluding early or late visual information. The authors found that providing participants with only early visual information (vision occluded on initiation of backswing) led to a significant detriment in performance, even when quiet eye durations were preserved. Conversely, when only late visual information (vision occluded from initial putter placement until initiation of backswing, when vision was returned) was available, there were no deficits in performance. These data support the role of online control during the quiet eye period and also provide evidence against a strict interpretation of quiet eye in which only preprograming of action is performed. However, it remains unknown whether the effects of occlusion can be negated if participants know in advance what visual information will be available, thus providing the opportunity to strategically adapt phases of both the quiet eye period and movement control. For example, it has been shown that in manual aiming movements, when an individual knows they will receive visual feedback they reduce movement planning (preprograming) time as they prepare to utilise vision for online control (Hansen et al. 2006; Khan et al. 2002).

The aim of the current study was to further examine the underlying processes of the quiet eye period by manipulating the duration that visual information was available for during the online control phase in a precision aiming task. Participants were required to complete a golf putting task to two different distances under two vision conditions: full vision and occluded vision. In the occluded vision condition, a liquid crystal (LC) panel turned opaque on initiation of the backswing, thus removing vision of the moving putter and stationary ball. The LC panel returned to the transparent state upon ball contact, thus providing vision of the ball trajectory. Two distances were used to determine the influence of complexity on preprograming and whether it is influenced by the lack on online visual feedback.

Based on previous research, it was predicted that overall quiet eye duration would be longer during putts with low radial error (low-RE), compared to putts with high radial error (high-RE) (Vickers 2007; Wilson et al. 2015). For the subcomponents of the quiet eye period, no outcome-related differences were predicted for QE-preprograming (Vine et al. 2013), whereas longer durations of QE-online (Vine et al. 2013) and QE-dwell (Vickers 1992, 2004, 2007; Vine et al. 2013) were predicted for low-RE compared to high-RE putts. With regard to the role of preprograming, it is predicted that the longer putts will require longer QE-preprograming duration compared to shorter putts (Williams et al. 2002). If preprograming alone occurs during the quiet eye period, we would expect no differences in outcome, or any changes in QE-online or QE-dwell between the two vision conditions. Conversely, if online visual control that would normally take place during the quiet eye period is eliminated by removing vision after movement onset, it can be expected that participants will adapt by increasing the duration of the QE-preprograming phase (Hansen et al. 2006; Khan et al. 2002). However, if online control is necessary during golf putting, it follows that a change in duration of QE-preprograming will not be sufficient to maintain performance accuracy in the occluded vision condition.

## Method

### Participants

Participants were 21 undergraduate students (*M* age = 23.3 years, SD = 4.5) who were novice golfers with no competition experience in the sport. All participants had normal to corrected normal vision and were right-handed. Written informed consent was obtained from the participants prior to participation. The research was conducted in accordance with the ethical guidelines of the lead institution.

### Task and apparatus

Visual search behaviours were recorded using a mobile corneal reflection system (Applied Science Laboratories; Waltham, MA, Model ASL Mobile Eye II). This video-based monocular system measures eye-line gaze using a head-mounted camera, by synchronising relative positions of both the corneal reflection (reflection of near-infrared light source from the surface of the cornea) and the pupil, in relation to the optics. The Mobile Eye has a system accuracy of 0.5° visual angle, resolution of 0.10° visual angle and visual range of 50° horizontal and 40° vertical.

**Fig. 1:**
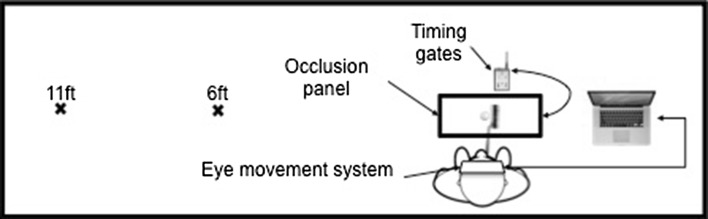
Schematic representation of experimental set-up

Figure 1 shows the experimental set-up. A Plato LC panel (Translucent Technologies, Toronto, Canada) was attached to a stand and linked to two infrared timing gates (Tag Heuer, Biel, Switzerland). The ball was placed in the centre on the field of view underneath the panel. The putter was aligned to the timing gates to ensure the panel turned opaque on initiation of the backswing. The panel became transparent on ball contact. Due to the arrangement of the screen and the location of the camera on the ASL Mobile Eye system, the camera could still see the ball when the screen occluded, whereas the participant was unable to see around the screen.

### Procedure

Participants were fitted with the Mobile Eye system, which was calibrated using a 9-point reference grid while participants were in their ‘normal’ golf stance. The Mobile Eye system recorded data for the entire duration of the test session with the accuracy of the calibration being monitored through a live feed.

Participants were required to make straight putts to two distances, 6-ft (1.83 m) and 11-ft (3.35 m), which were marked on a putting surface.^1^ All participants used a standardised putter and a standard size white golf ball. Participants were required to putt in a full vision condition, and an occluded vision in which the LC panel turned opaque on initiation of backswing. For familiarisation, participants completed 10 practice putts to a 9-ft target. Next, participants completed 80 experimental putts (8 blocks of 10), consisting of 20 putts for each combination of target distance (6-ft, 11-ft) and vision condition (full vision and occluded vision). Distance was randomised, but vision condition was blocked. The distance and vision conditions were randomised between participants. The experiment lasted for approximately 60 min.

### Measures

*Radial error* was recorded as the measure of putting performance and was defined as the Euclidean distance the ball finished from the hole in mm.

*Quiet eye duration* was defined as the final fixation, within 1° of visual angle for a minimum of 100 ms, towards the ball before the initiation of the backswing (Vickers 2007; Vine et al. 2013). The onset of quiet eye was defined as the initiation of the final fixation that occurred before the start of the backswing and was marked by the first frame in which the performer directed their gaze towards the ball. The offset of quiet eye occurred when the gaze deviated from the ball location by 1° of visual angle for more than 100 ms (Vickers 2007). Due to the placement of the camera on the ASL Mobile Eye, the ball was still in view of the camera when the screen occluded; the participants could not see the ball at this time.

*Preprograming duration* was defined as the component of the quiet eye starting at quiet eye onset and ending with the initiation of the backswing. As such, this duration reflects the proportion of the quiet eye that may be responsible for the preprograming of the ensuing putting stroke (Vine et al. 2013).

*Online duration* was defined as the component of the quiet eye starting with the initiation of the backswing and finishing when the putter contacted the ball, or when gaze deviated from the ball by 1° of visual angle for more than three frames. As such, this duration reflects the proportion of the quiet eye that may be largely responsible for the online control of the putting stroke (Vine et al. 2013).

*Dwell duration* was defined as the component of the quiet eye that started when the putter contacted the ball and ended when the gaze deviated from the same location on the green by 1° of visual angle for more than three frames (Vickers 2007). If the quiet eye offset occurred before ball–putter contact, then dwell was recorded as zero.

*Movement phase durations* were recorded for the preparation, backswing and foreswing of the putting action. Preparation was defined as the moment when the ball was addressed until the initiation of backswing. Backswing was defined as the initiation of backswing until the clubhead stopped its backward motion. Foreswing was defined as the first forward motion of the clubhead until ball contact (Vickers 2007). Trials where the ball was not visible at club–ball contact were discarded.

### Statistical analysis

The data were coded using the Quiet Eye Solutions software (Calgary, CA), which couples automatically the gaze and kinematics. For each participant, a median split of radial error scores was used to separate two outcome groups: low-RE and high-RE (see Table 1). This enabled differences in all dependent variables to be compared between more accurate and less accurate trials. Radial error and all quiet eye variables were submitted to separate ANOVA with vision condition (full vision, occluded vision), distance (6-ft, 11-ft) and outcome (low-RE, high-RE) as within-participants factors. Phase durations were also analysed using ANOVA with movement phase (preparation, backswing, foreswing), vision condition (full vision, occluded vision), distance (6-ft, 11-ft) and outcome (low-RE, high-RE) as within-participants factors. The alpha level for significance was set at 0.05.

**Table 1:** Mean radial error (mm) for each participant in the occlusion and full vision conditions for 6-ft and 11-ft putts in low radial error (low-RE) and high radial error (high-RE) groups

Participant	Occlusion				Full vision			
	6-ft		11-ft		6-ft		11-ft	
	Low-RE	High-RE	Low-RE	High-RE	Low-RE	High-RE	Low-RE	High-RE
1	155	643	315	1075	165	579	226	724
2	200	384	325	779	128	316	317	763
3	181	646	369	977	160	375	273	661
4	190	541	239	966	129	419	186	622
5	131	466	295	1074	104	409	214	660
6	149	360	190	665	103	337	232	532
7	294	939	388	1219	164	548	422	1038
8	167	541	159	752	106	371	353	848
9	167	712	286	892	148	389	372	914
10	169	520	393	1089	160	470	242	824
11	195	375	381	801	119	296	346	736
12	193	577	407	1091	192	375	320	703
13	160	446	272	1044	102	322	180	553
14	115	437	362	1220	122	421	263	692
15	144	344	198	745	85	318	233	593
16	300	915	432	1192	203	672	504	1220
17	179	535	157	759	102	392	346	846
18	234	771	329	923	147	401	274	885
19	196	362	429	857	125	263	340	728
20	231	630	340	883	153	335	326	703
21	178	581	227	993	137	383	176	479
Mean	187	558	309	952	136	400	293	749

## Results

### Radial error

**Fig. 2:**
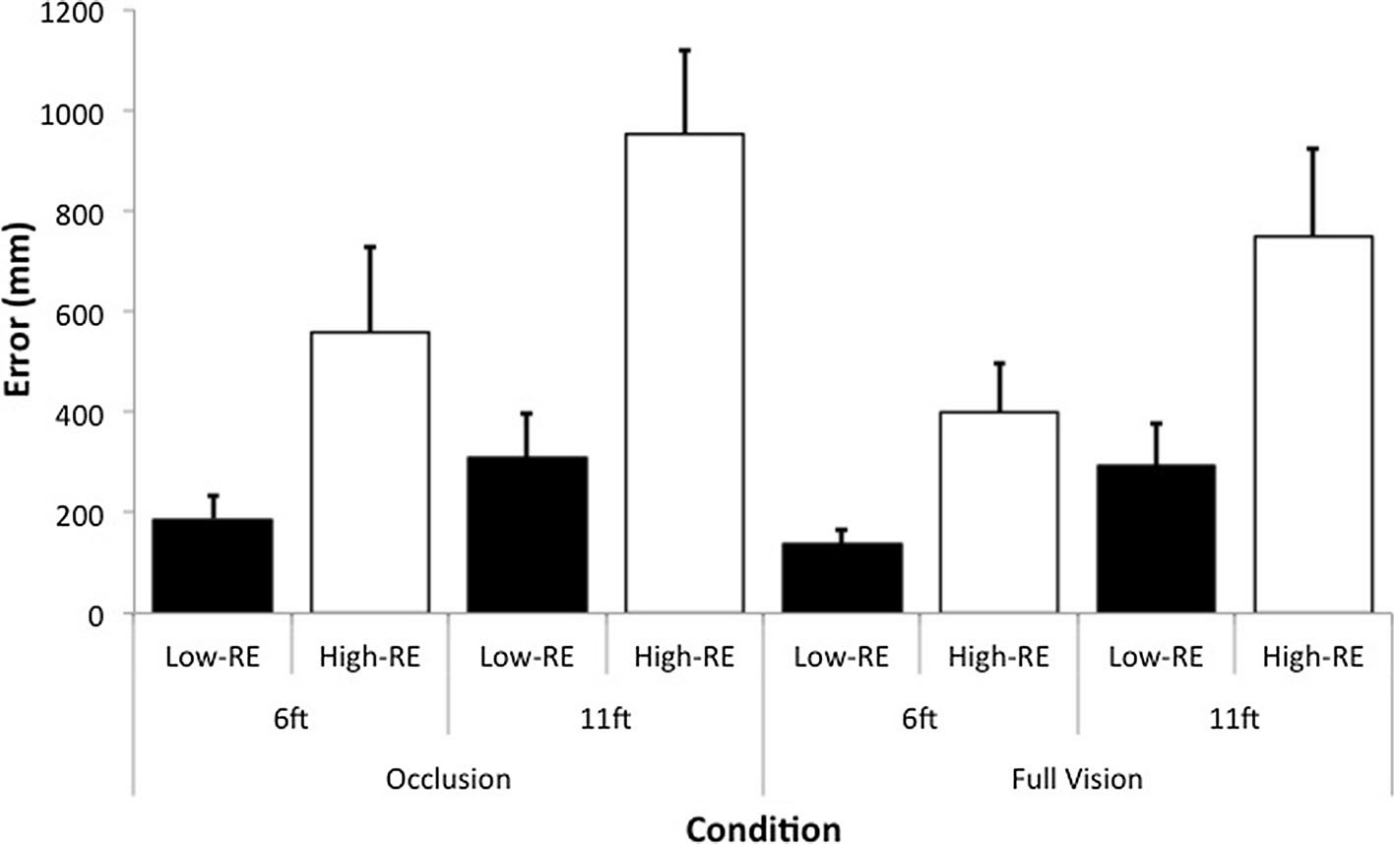
Mean (SD) radial error (mm) for the two vision conditions (occluded vision, full vision), two distances (6-ft, 11-ft) and two outcomes (low-RE, high-RE)

A main effect was found for outcome *F*(1, 20) = 417.69, *p* < .001, with greater radial error exhibited in high-RE trials (*M* = 664.71 mm, SD = 258.23), compared to low-RE trials (*M* = 231.19 m, SD = 98.84). There were also main effects for vision *F*(1, 20) = 57.51, *p* < .001, and distance *F*(1, 20) = 401.57, *p* < .001. These were superseded by interactions between vision and outcome *F*(1, 20) = 61.88, *p* < .001, and distance and outcome *F*(1, 20) = 430.25, *p* < .001 (see Fig. 2). There was a significantly larger increase in radial error between high-RE and low-RE trials for the occluded vision, compared to the full vision condition. Also, there was a significantly larger increase in radial error between high-RE and low-RE trials for the longer, compared to the shorter putts. There was no vision, distance and outcome interaction *F*(1, 20) = 3.84, *p* = .064.

### Quiet eye duration

A main effect was found for outcome *F*(1, 20) = 19.21, *p* < .001, with longer quiet eye duration in low-RE trials (*M* = 1183.93 ms, SD = 372.68), compared to high-RE trials (*M* = 844.64 ms, SD = 293.67). A main effect was found for vision *F*(1, 20) = 13.10, *p* < .001, with quiet eye duration being longer in the occluded vision condition (*M* = 1095.48 ms, SD = 365.14), compared to the full vision condition (*M* = 933.10 ms, SD = 369.74). A main effect was found for distance *F*(1, 20) = 53.41, *p* < .001, with quiet eye duration being longer on the 11-ft putts (*M* = 1134.29 ms, SD = 360.08), compared to the 6-ft putts (*M* = 894.29 ms, SD = 352.95). There were no two- or three-way interactions concerning vision, distance and outcome (all *ps* > .05).

### Preprograming duration

A main effect for outcome *F*(1, 20) = 9.91, *p* = .005, indicated preprograming duration was longer in low-RE trials (*M* = 860.67 ms, SD = 430.09) compared to high-RE trials (*M* = 621.35 ms, SD = 353.27). Main effects were also found for vision *F*(1, 20) = 102.77, *p* < .001, and distance *F*(1, 20) = 53.49, *p* < .001. Preprograming duration was longer in the occluded vision condition (*M* = 962.13 ms, SD = 358.23), than the full vision condition (*M* = 519.88 ms, SD = 333.89), and on 11-ft putts (*M* = 859.85 ms, SD = 394.60), compared to 6-ft putts (*M* = 622.17 ms, SD = 393.04). There were no two- or three-way interactions concerning vision, distance and outcome (all *ps* > .05).

### Online duration

**Fig. 3:**
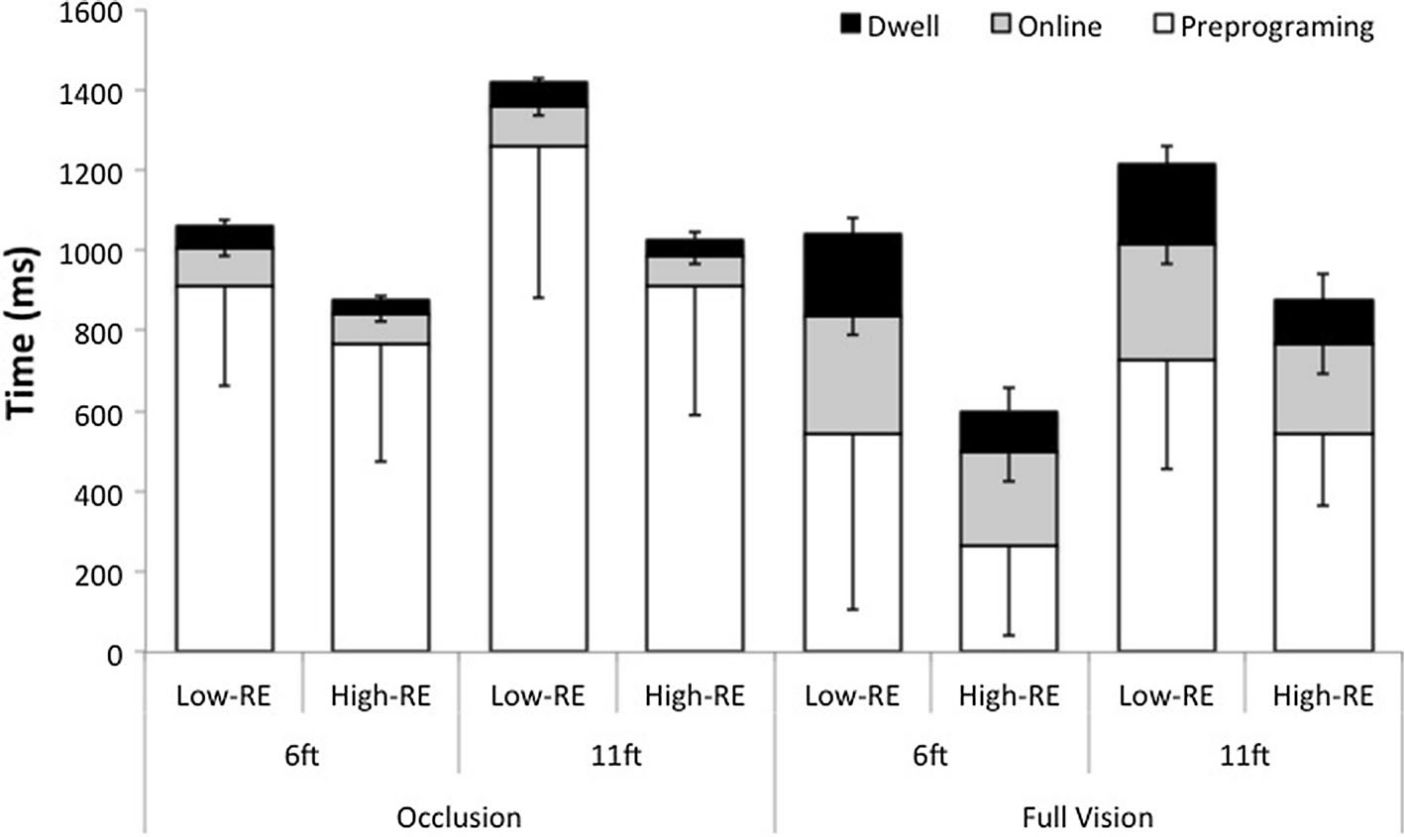
Mean (SD) preprograming, online and dwell durations (ms) for the two vision conditions (occluded vision, full vision), two distances (6-ft, 11-ft) and two outcomes (low-RE, high-RE)

A main effect for outcome *F*(1, 20) = 21.50, *p* < .001, indicated a longer online duration was exhibited in low-RE trials (*M* = 192.76 ms, SD = 105.68), compared to high-RE trials (*M* = 151.38 ms, SD = 93.09). A main effect of vision *F*(1, 20) = 428.47, *p* < .001, indicated online duration was shorter in the occluded vision condition (*M* = 84.69 ms, SD = 21.66), compared to the full vision condition (*M* = 295.45 ms, SD = 60.07). There was a vision and outcome interaction *F*(1, 20) = 8.50, *p* = .009, which indicated a larger decrease in online duration between high-RE and low-RE trials for the full vision, compared to the occluded vision condition (see Fig. 3). There were no two- or three-way interactions concerning vision, distance and outcome (all *ps* > .05).

### Dwell duration

A main effect for outcome *F*(1, 20) = 108.68, *p* < .001, indicated dwell duration was longer in low-RE trials (*M* = 130.41 ms, SD = 77.25), compared to high-RE trials (*M* = 71.94 ms, SD = 55.16). A main effect for vision *F*(1, 20) = 273.53, *p* < .001, indicated dwell duration was shorter in the occluded vision condition (*M* = 48.64 ms, SD = 17.30), compared to the full vision condition (*M* = 153.70 ms, SD = 69.72). A significant vision and outcome interaction *F*(1, 20) = 52.99, *p* < .001, showed a greater decrease in dwell duration during full vision, compared to the occluded vision condition in high-RE and low-RE trials. There were no two- or three-way interactions concerning vision, distance and outcome (all *ps* > .05).

### Movement phase durations

A main effect of outcome *F*(1, 20) = 5.08, *p* = .035, indicated movement phase durations were significantly shorter in high-RE trials (*M* = 1242.68 ms, SD = 1322.99), compared to low-RE trials (*M* = 1325.11 ms, SD = 1403.58). A main effect for vision *F*(1, 20) = 121.96, *p* < .001, indicated movement phase durations were significantly shorter in the full vision condition (*M* = 1090.46 ms, SD = 1599.58), compared to the occluded vision condition (*M* = 1477.32 ms, SD = 1044.05). A main effect for movement phase, *F*(2, 40) = 2340.44, *p* < .001, indicated movement time in the preparation phase (*M* = 3052.17 ms, SD = 918.91) was longer than both the backswing (*M* = 457.52 ms, SD = 138.11) and foreswing phases (*M* = 341.99 ms, SD = 91.24). The backswing phase was also longer (*p* < .05) than the foreswing phase. An interaction between vision and movement phase *F*(2, 40) = 138.92, *p* < .001, showed movement time in the preparation phase was longer in the occluded vision condition compared to the full vision condition (see Table 2). There was no difference in movement time between the vision conditions in the backswing or foreswing phases. All other interactions were not significant (all *ps* > .05).

**Table 2:** Mean (SD) movement phase durations (ms) for the preparation, backswing and foreswing phases in the occlusion and full vision conditions for 6-ft and 11-ft putts in low radial error (low-RE) and high radial error (high-RE) groups

Movement phase	Occlusion				Full vision			
	6-ft		11-ft		6-ft		11-ft	
	Low-RE	High-RE	Low-RE	High-RE	Low-RE	High-RE	Low-RE	High-RE
Preparation	3761 ± 752	3551 ± 663	3823 ± 510	3443 ± 1033	2433 ± 634	2472 ± 643	2626 ± 665	2310 ± 602
Backswing	433 ± 87	428 ± 93	454 ± 120	494 ± 84	429 ± 144	363 ± 120	533 ± 184	526 ± 169
Foreswing	361 ± 83	304 ± 67	351 ± 84	326 ± 54	366 ± 67	290 ± 107	333 ± 79	407 ± 126

## Discussion

The quiet eye period has been associated with cortical organisation and preprograming of movement parameters (i.e. force, velocity and direction) that are required for skill execution (Vickers 1996a, b, c). By prioritising task-relevant visuo-spatial information for skill execution during the final fixation, cortical resources are less likely to be allocated to analytical processing and irrelevant sensory cues (Vickers 2009). Consequently, longer QE duration can result in more efficient movement kinematics requiring fewer online corrections (Causer et al. 2010, 2011), and more accurate performance (Williams et al. 2002). There is also evidence suggesting processing associated with online visual control can occur in the quiet eye period (Vine et al. 2013, 2015). The current study was designed to examine the respective roles of preprograming and online control during the quiet eye period by manipulating visual information available in golf putting. To this end, we modified the protocol used by Vine et al. (2015), who found that randomly removing access to late information, but not early information, had a significant impact on putting performance. Here, we required novice participants to putt at a target located at two different distances, with vision available throughout or occluded at the initiation of the backswing such that the moving putter and stationary ball could no longer be seen. In addition, following the procedures reported by (see Vine et al. 2013), we split the quiet eye period into subsections in order to determine if gaze, and thereby information processing, was modulated by the experimental manipulations.

Having performed a median split of radial error (RE) scores for each participant (Vickers 1996c), we found that low-RE trials were associated with longer quiet eye durations, thus providing construct validity to the task, as well as corroborating findings from multiple aiming experiments and interceptive tasks (for reviews see Gonzalez et al. 2015; Wilson et al. 2015). The durations of each phase of the quiet eye period (preprograming, online and dwell) were also longer in low-RE compared to high-RE trials (Vine et al. 2013). In addition, radial error was significantly larger in the 11-ft compared to the 6-ft putt, and in the occluded vision condition compared to the full vision condition.

A similar pattern of results to those of RE was observed for quiet eye duration, which was longer in the 11-ft compared to the 6-ft distance, as well as in the occluded vision compared to full vision condition. The increase in quiet eye duration during the occluded vision condition suggests participants strategically adapted to the restricted availability of vision after backswing initiation, and hence the lack of visual information for online control during the putt, by investing more time in the preprograming phase. This is also corroborated by the movement phase data showing a longer preparation phase in the occluded vision condition.

However, not influenced by putting distance, online duration and dwell time were longer in the vision condition compared to occluded vision condition. By maintaining gaze fixation on the ball for a longer duration after initiation of the backswing and during the follow-through (i.e. dwell time), it follows that participants could have taken advantage of peripheral and central visual information regarding the putter movement, and thereby reduced RE. These data are consistent with the suggestion that during putting experienced golfers constantly regulate the distance between club and ball and compare this to an internal model of the expected sensory consequences (Craig et al. 2000). Importantly, they also corroborate Vine et al. (2015)^2^ who found that occluding late, but not early, information significantly decreased performance outcome.

In the occluded vision condition in the present study, participants did not have access to vision for online control but there was still a potential advantage to maintain gaze fixation at the ball location because the LC panel once again became transparent at putter–ball contact. Why then, did fixation change after movement initiation? A reasonable explanation is that by occluding vision of the ball and surrounds, participants no longer had a visual target upon which to anchor gaze and thus would have been more prone to move their eyes during the online duration. In addition, soon after occlusion the eyes would have adopted the physiological position of rest rather than remaining converged on a memorised position of the ball. Accordingly, there would have been poor binocular fusion of the ball at the moment of contact, which could have been disruptive to online control.

Having found gaze fixation data indicative of processing associated with both preprograming and online control during the quiet eye period, the question remains how was this reflected in the golf putting movement. Of the three phases of golf putting, we found that only the duration of the preparation phase was increased in the occluded vision condition compared to the full vision condition. This is consistent with participants spending more time planning and programming the putting action when vision was not available for online control (Hansen et al. 2006; Khan et al. 2002). It is noteworthy that the increase in duration of the preparation phase was not sufficient to overcome the loss of visual information and thus maintenance of outcome performance accuracy. The duration of the backswing and foreswing phases were similar to previous work (Vine et al. 2013), and as expected were shorter than the duration of the preparation phase. Even though participants knew they would not have access to vision for online control, they did not exhibit a slowed and more deliberate action in the occluded vision condition. This is perhaps not surprising as golf putting is a dynamic action where it is necessary to impart a certain amount of force to the ball by the putter in order to ensure it reaches the intended target.

To conclude, we showed that increasing task difficulty and/or removing the availability of vision for online control increased quiet eye duration prior to movement initiation (Klapp 1977; Mann et al. 2011; Williams et al. 2002), as well as the duration of the movement preparation phase. These changes to ocular and sensorimotor control were reflective of a more successful outcome. Importantly, however, this strategic adaptation in quiet eye and sensorimotor control did not enable participants to maintain similarly accurate outcome performance to the vision condition, thus confirming the contribution of online visual control during the quiet eye period when golf putting (Oudejans et al. 2002; Vine et al. 2013, 2015; Wilson et al. 2015). Further research is required to develop an understanding of the specific mechanisms underpinning these quiet eye processes and how they can be manipulated to increase performance (Gonzalez et al. 2015).

## Footnotes

1 Aiming to a target rather than a hole has limitations, such as task representativeness, and the potential confound of combining hits and misses (Vickers 2016). However, due to the novice sample having no experience of either task, and the difficultly of gaining an equal number of hits and misses in all conditions, radial error was used to provide a sensitive measure of outcome.

2 It is worth noting that, compared to previous studies (Vine et al. 2013, 2015), the quiet eye durations are shorter in the current study. These differences can be attributed to the task constraints, such as: different distances, and thus differing processing demands; aiming at a target, rather than a hole; and the use of truly novice participants, compared to skilled or less-skilled participants in other studies (Vickers 1992, 2004; Vine et al. 2013, 2015).
